# Transesophageal Echocardiography Improves Precision in Transseptal Puncture Compared to Fluoroscopy in Left Atrial Electrophysiological Procedures

**DOI:** 10.3390/jcm13092476

**Published:** 2024-04-24

**Authors:** Lyuboslav Katov, Yannick Teumer, Katrin Lederbogen, Rima Melnic, Wolfgang Rottbauer, Carlo Bothner, Karolina Weinmann-Emhardt

**Affiliations:** Department of Cardiology, Ulm University Heart Center, Albert-Einstein-Allee 23, 89081 Ulm, Germanywolfgang.rottbauer@uniklinik-ulm.de (W.R.);

**Keywords:** transseptal puncture, electrophysiology, pulmonary vein isolation, transesophageal echocardiography, fluoroscopy

## Abstract

**Background:** Complex arrhythmias often arise from the left side of the heart, necessitating established electrophysiological (EP) procedures like 3D-mapping-assisted radiofrequency (RF) ablations or pulmonary vein isolation (PVI). These procedures typically require transseptal access, emphasizing the critical role of achieving an optimal catheter position through a precise transseptal puncture (TSP). Commonly employed imaging methods for TSP guidance include fluoroscopy and interventional echocardiography. Despite their routine use, there is limited evidence on which imaging modality offers superior catheter positioning for EP procedures, and safety concerns regarding transseptal punctures with imaging remain underexplored. This study aims to systematically evaluate the feasibility, safety, and accuracy of echo-guided TSP compared to fluoroscopy-guided TSP. **Methods:** In this prospective study, 150 consecutive patients undergoing left atrial EP procedures were enrolled between October 2023 and February 2024 at the Ulm University Heart Center. Following optimal fluoroscopy-guided transseptal needle positioning at the interatrial septum, the catheter placement was further verified using transesophageal echocardiography (TEE). Adjustments were made in cases of suboptimal needle positioning observed in TEE. The fluoroscopically achieved septal positions were categorized based on TEE images as optimal, suboptimal, poor, or dangerous. **Results:** Among the 150 patients included (58.0% male), fluoroscopy achieved optimal, suboptimal, and poor/dangerous positions in 32.7%, 43.3%, and 24.0%, respectively. After TEE-guided adjustments, optimal and suboptimal positions were achieved in 59.3% and 40.7% of patients, respectively. No instances of poor or dangerous transseptal needle positions were observed under TEE guidance. **Conclusions:** TEE-guided TSP emerges as a feasible, more accurate, and safer imaging method for transseptal punctures in EP procedures.

## 1. Introduction

Complex arrhythmias often originate from the left side of the heart, with atrial fibrillation (AF) standing out as the most prevalent. The prevalence of arrhythmias is increasing due to an aging population. The estimated lifetime risk of AF is 33% [1]. Pulmonary vein isolation (PVI) has evolved as the cornerstone of AF treatment, and in the attempt at early rhythm control, symptomatic patients are considered for ablation in an early stage of the disease’s course. Thus, AF ablation has become a standard procedure [1,2,3]. Furthermore, AF is predisposed to the occurrence of additional left atrial tachycardias, such as focal atrial tachycardias and atrial atypical flutters, which may also arise as a consequence of a proarrhythmic effect subsequent to PVI [4,5]. In particular, left atrial flutters have a greater prevalence [6,7].

Over the last two decades, numerous studies have demonstrated the effectiveness of percutaneous catheter-based ablation as an approach to treating cardiac arrhythmias [2,8,9,10]. To be able to perform such an ablation for tachycardias originating from the left atrium, one or more punctures via the interatrial septum are almost always necessary. This technique of a transseptal puncture (TSP) was developed in 1958 for diagnostic purposes in left heart catheterization and to measure left atrial pressure. Commercially available sheaths and ablation systems are designed to access the left atrium via TSP. Therefore, every interventional electrophysiologist should be well-trained in this technique to guarantee adequate and safe access through the interatrial septum [11,12,13].

An optimal location of the TSP is a prerequisite to achieving an optimal position of the catheters during the electrophysiological (EP) procedure. Depending on the ablation system used, a different position at the septum must be chosen for the TSP to ensure optimal deployment and success of the intervention. To be able to perform the TSP safely, interventionalists require a thorough anatomical understanding of the interatrial septum and the surrounding structures [13,14]. Furthermore, there are several safety mechanisms available to mitigate complications. A critical structure near the interatrial septum is the aortic root. During a TSP for left heart catheterization, a pig-tail catheter is frequently positioned at the aortic root as a landmark to avoid an unintended puncture of the aorta [15,16]. In EP procedures, electrode catheters such as the His bundle and the coronary sinus catheter can serve as additional landmarks. These catheters can also help modify the angles of the right and left anterior oblique projections according to the individual rotation of the patient’s heart. Additionally, the electrodes recording proximal His bundle activation correspond with the localization of the aortic root [15]. Given that each heart chamber exhibits a characteristic pressure curve, pressure monitoring at the needle tip of the TSP system allows the investigator to determine the heart compartment entered by the needle following the puncture. In case of a misguided puncture, it is possible to retract the needle before advancing the TSP system. Moreover, one can introduce a thin guidewire through the lumen of the TSP needle after the puncture and advance the wire to a pulmonary vein to confirm access to the left atrium. Additionally, there is a need for an appropriate imaging technique to find the perfect point at the interatrial septum for the TSP [17]. Mainly, only fluoroscopy is used to locate the optimal point on the interatrial septum for the TSP [17]. An alternative imagining technique is interventional transesophageal or intracardiac echocardiography (TEE/ICE) [17]. Despite its frequent use, there is limited evidence regarding which imaging method for TSP leads to a better catheter position for EP procedures. Furthermore, there is limited evidence regarding the safety of punctures in relation to imaging. Consequently, the aim of this study was to evaluate the feasibility, safety, and accuracy of a TEE-guided TSP in comparison to the fluoroscopy-guided TSP in routine use in the EP laboratory.

## 2. Materials and Methods

### 2.1. Study Population

In our prospective study, we enrolled 150 consecutive patients who underwent left atrial EP procedures at Ulm University Heart Center between October 2023 and February 2024. Inclusion criteria were left atrial arrhythmias, such as AF, left atrial flutters, and focal left atrial tachycardia, requiring TSP access for ablation. Patients with left ventricular arrhythmias requiring TSP were excluded. Furthermore, patients with pre-existing atrial septal defects, often resulting from a pre-ceding left atrial intervention, or those with a persistent foramen ovale, which might have been inadvertently probed, were not included in the study. Each patient gave written informed consent prior to the procedure. This study was approved by the Ethics Committee of Ulm University and conforms with the principles outlined in the Declaration of Helsinki. The data were collected prospectively as part of the ATRIUM registry (German Clinical Trials Register-ID: DRKS00013013).

### 2.2. Sedation Protocol and Transesophageal Echocardiography Probe Placement

Deep sedation of the patient was achieved with an initial bolus of midazolam for anxiolysis and continuous administration of propofol for sedation in combination with an opioid. In the case of cryo-balloon PVI and pulsed-field ablation, a fentanyl bolus was administered after TSP. In the 3D-mapping procedures, remifentanil was administered continuously via a perfusor. The detailed sedation protocol was described previously [18].

The TEE was performed by an experienced examiner. The TEE probe was introduced with the patient in a supine position with their face flexed towards the TEE examiner directly after deep sedation was achieved. If multiple attempts for TEE probe placement failed, a laryngoscope was utilized to introduce the TEE under sight. The required attempts for intubation with the TEE probe, their duration, and the total in-body time of the TEE probe were documented. Furthermore, associated major complications and minor complications were documented. Major complications we considered were severe mucosal bleeding requiring further intervention, tooth damage, perforations, or hematomas requiring surgical treatment. Minor complications were defined as mild mucosal bleeding and hematomas, which did not require intervention.

### 2.3. Transseptal Puncture Protocol

First, the left atrial thrombus was ruled out via TEE. The procedure commenced with two venous punctures in the right groin, establishing access for subsequent navigation to the right atrium. Subsequently, a steerable 10-polar catheter (Inquiry™ Steerable Diagnostic Catheter, Abbott, North Chicago, IL, USA) was placed in the coronary sinus. This catheter served a dual purpose—recording signals from the coronary sinus and providing visual guidance for the subsequent TSP. As a second step, a non-steerable transseptal sheath (CardiaGuide™, Johnson & Johnson, New Brunswick, NJ, USA) was introduced into the superior vena cava via a guidewire. Afterward, a transseptal needle (HeartSpan™ Transseptal Needle™, Johnson & Johnson, New Brunswick, NJ, USA) was introduced via the inner lumen of the non-steerable transseptal sheath. The transseptal needle was equipped with a pressure measurement system. Following the preparation of the TSP system, which included the transseptal sheath and needle, the TSP system was carefully retracted under fluoroscopic guidance only. Thereby, the apex of the TSP system was orientated to the interatrial septum. The apex of the TSP system was retracted first to the right atrium and then precisely to the fossa ovalis. The examiner could thereby observe two jumps of the TSP system fluoroscopically: the first jump is due to the entry of the TSP system from the superior vena cava into the right atrium, and the second jump is due to the transition from the apex of the TSP system from the ‘normal’ interatrial septum into the fossa ovalis. The transition of the TSP system into the right atrium was observed from a left anterior oblique (LAO) 40° or anteroposterior (AP) 0° position, which also facilitated determining the height of the TSP system in this view. The anterior–posterior position was determined fluoroscopically in a right anterior oblique (RAO) 30° view.

**Figure 1:**
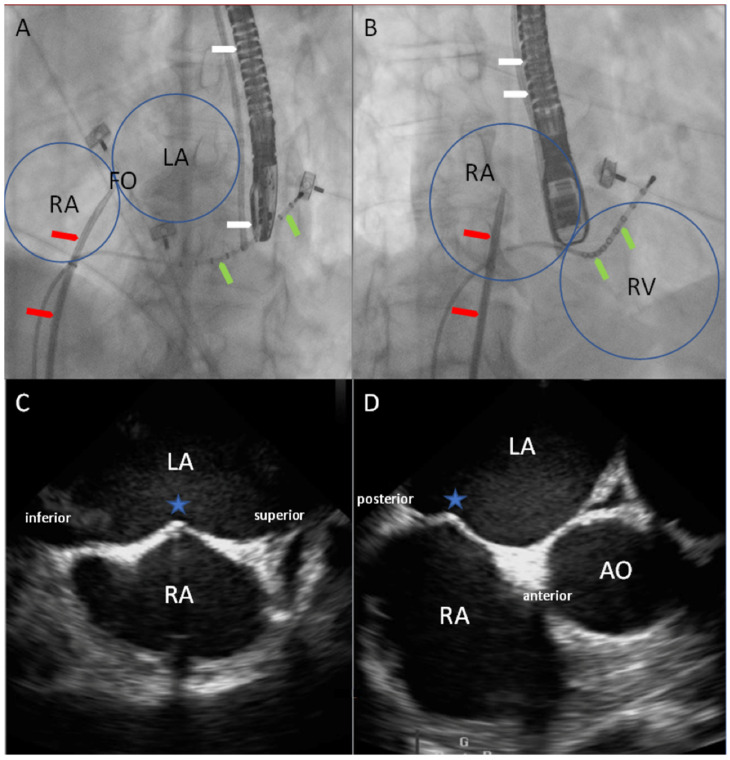
Anatomical boundaries of the heart chambers under fluoroscopy (**A**,**B**) and under TEE (**C**,**D**). Schematic illustration of the heart under fluoroscopy (**A**,**B**) and under TEE (**C**,**D**). On the upper left side (**A**), a left anterior oblique (LAO) 40° view can be seen, and on the upper right side (**B**), a right anterior oblique (RAO) 30° view. Besides the anatomical relations between the right and left heart chambers, the positions of the coronary sinus catheter (green arrows), the fossa ovalis, and the transseptal puncture system (red arrows) in relation to the aforementioned structures can be observed. In the middle, the TEE probe can be seen (white arrows). Correspondingly, the mid-transesophageal view at 135° (**C**), and at 45° (**D**). The blue stars indicate the tenting position of the TSP system. AO, aorta; FO, fossa ovalis; LA, left atrium; RA, right atrium; RV, right ventricle.

After optimal fluoroscopic placement of the TSP system, the images of the parallel performed TEE were unblinded for the TSP-performing interventionalist and adjusted to the best possible location, if necessary. In general, the position of the TSP system was determined via TEE (Philips CX50 ultrasound system, in combination with a Philips X7 TEE probe, Philips, Amsterdam, The Netherlands) from a mid-transesophageal position with simultaneous visualization of orthogonal views (views at 45° and 135°, Figure 1). The standard protocol consisted of obtaining a midesophageal aortic valve short-axis view at 45°, with a focus on the interatrial septum. In this view, we assessed the anterior/posterior position, paying particular attention to the fossa ovalis. The corresponding orthogonal view was a modified midesophageal bicaval view at 135°, with emphasis on the needle tip at the septum. In this view, we evaluated the inferior/superior dimension of the TSP system. Both views were displayed simultaneously for perfect TSP system placement. When the best possible position was achieved at the interatrial septum, the advancement of the needle was observed under TEE control. The final position of the TSP was documented. 

The fluoroscopically achieved septal position of the TSP system was classified by three investigators as optimal, suboptimal, poor, or dangerous. 

**Figure 2:**
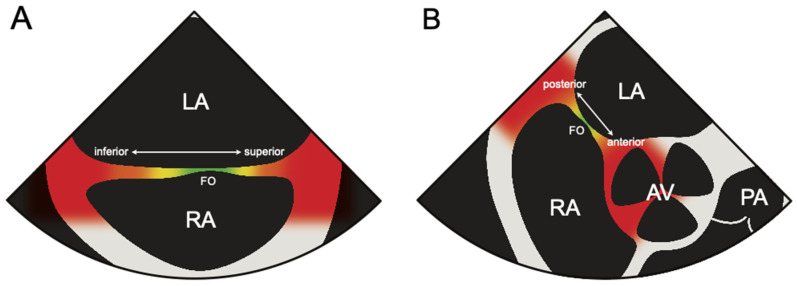
Depiction of the transseptal puncture (TSP) system positioning achieved fluoroscopically was reevaluated under transesophageal echocardiography (TEE) guidance. (**A**) Schematic representation of a modified midesophageal bicaval view at 135° of the interatrial septum. The fluoroscopically achieved positions are categorized and color-coded as follows: green for optimal, yellow for suboptimal, orange for poor, and red for dangerous regarding the inferior/superior position. (**B**) Schematic representation of the midesophageal aortic valve short-axis view at 45° of the interatrial septum, with the thin section indicating the fossa ovalis (FO). The same color code as above is used here to represent the fluoroscopically achieved positions regarding the anterior/posterior position. AV, aortic valve; FO, fossa ovalis; LA, left atrium; PA, pulmonary artery; RA, right atrium.

The optimal position was defined as the most suitable for the planned intervention and ablation method (Table 1). The suboptimal positioning of the TSP system enabled a safe puncture, although it may have had potentially mild restrictions on the further progression of the procedure due to things like limited catheter movement. The poor positioning of the TSP system would enable a safe puncture but significantly impact the execution of the procedure. The dangerous position was defined as a placement of the TSP system that risks cannulation of an anatomical structure other than the left atrium, such as the aorta or pericardium (Figure 2). The position of the TSP system, which was classified as dangerous, was always corrected under TEE control to an at least suboptimal position and, if possible, to an optimal position. A detailed description of all possible positions of the TSP system is presented in Table 1.

After achieving the best possible placement under TEE guidance, the TSP was performed. After the transseptal needle passed the interatrial septum, the left atrial pressure and graph were documented. Afterward, a coronary guidewire (Balance Heavy Weight, Abbott, North Chicago, NJ, USA) was introduced through the inner lumen of the transseptal needle into the left atrium with the aim of probing the left upper pulmonary vein. As the next step, the TSP system was advanced via this guidewire to the left superior pulmonary vein. The transseptal sheath was subsequently replaced with a steerable transseptal sheath (for the RF-PVI: Vizigo™ steerable sheath, 8.5F, Biosense Webster, Irvine, CA, USA or Agilis NxT steerable sheath, 8.5F, Boston Scientific, Marlborough, MA, USA; for the pulsed-field ablation (PFA) PVI: Faradrive™ steerable sheath, 13F, Boston Scientific, Marlborough, MA, USA; for the CB PVI: Polarsheath™ steerable sheath, 12.7F, Boston Scientific, Marlborough, MA, USA or FlexCath™ Advance steerable sheath, 12F, Medtronic, Dublin, Ireland). A pericardial tamponade was ruled out before the TEE probe was removed from the patient.

### 2.4. Statistical Analysis

Statistical analyses were performed using Excel^®^ (version 16.45, Microsoft Corporation, Washington, DC, USA) and SPSS^®^ Statistics (version 29.0.1.0, IBM, Armonk, NY, USA). Variables were analyzed depending on the scale level. Categorical variables were depicted using absolute and relative proportions. Continuous variables were expressed as mean ± standard deviation. A *p*-value < 0.05 was considered statistically significant. The different TSP system positions were assessed using the Chi-square test.

## 3. Results

### 3.1. Study Population

In total, 150 patients were enrolled. The average age of the patients was 71 ± 12 years. A total of 87 patients (58.0%) were male. The mean body mass index (BMI) was 27.4 ± 4.7. The mean left ventricular ejection fraction was 53 ± 13%, and the mean left atrial diameter was 4.6 ± 0.7 cm. Detailed baseline characteristics are shown in Table 2.

### 3.2. Procedural Characteristics

In 75 patients (50%), 3D-mapping-guided RF ablation was performed; in 38 patients (25.3%), a cryo-balloon (CB) PVI was performed; and in 37 patients (24.7%), PFA-PVI was performed. The mean procedure duration was 99 ± 45 min. The fluoroscopy time was 15.0 ± 8.3 min (Table 3).

The mean number of attempts for intubation with the transesophageal probe was 1.8 ± 1.5 times, and the mean in-body time of the TEE was 17 ± 8 min. In 94 procedures (62.7%), the TEE probe was placed in the first attempt. In two procedures (1.3%), the transesophageal probe could not be introduced due to anatomical abnormalities; the time loss due to unsuccessful TEE intubation was 30 min in the first case and 25 min in the second case. One patient (0.7%) experienced a pharyngeal hematoma without further sequelae because of a complicated TEE probe placement. Detailed procedural data regarding the TEE are provided in Table 4. In this cohort, no complications associated with the TSP, especially pericardial effusion or tamponade, were observed.

### 3.3. Position of the TSP System under Fluoroscopy and TEE Guidance

Exclusively guided by fluoroscopy and subsequently evaluated using TEE, an optimal position of the TSP system was achieved in 49 patients (32.7%), a suboptimal position in 65 patients (43.3%), and a poor or dangerous position in 12 patients (8.0%) and 24 patients (16.0%), respectively.

**Figure 3:**
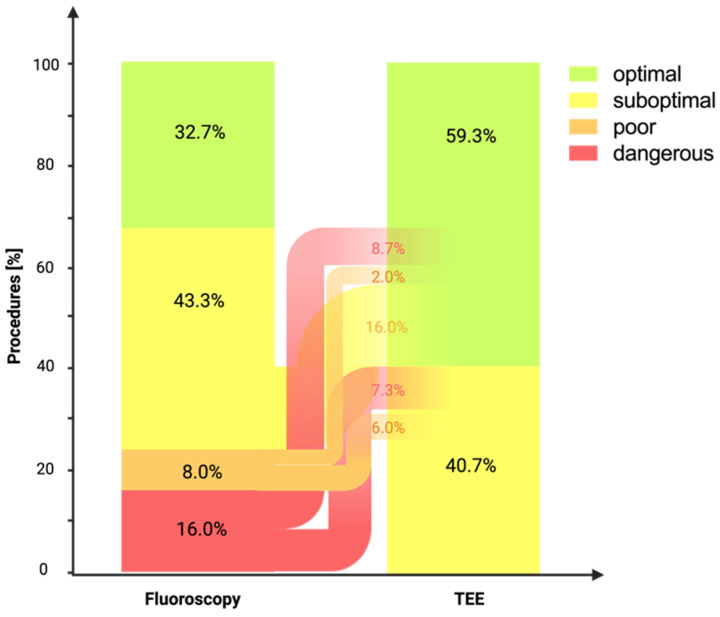
Best position of the transseptal puncture system under fluoroscopic guidance and after TEE adjustment. Demonstration of the results from the positioning of the TSP system under solely fluoroscopic guidance (left column) and subsequently after TEE adjustment (right column). Under fluoroscopy, four positions were revealed following TEE assessment—optimal (green), suboptimal (yellow), poor (orange), and dangerous (red), while with the help of TEE adjustment, only optimal (green) and suboptimal (yellow) positions were observed. TEE, transesophageal echocardiography.

No correction was necessary for patients for whom an optimal position of the TSP system was achieved under fluoroscopic guidance (*n* = 49, 32.7%). In patients in whom a suboptimal placement (*n* = 65, 43.3%) of the TSP system under fluoroscopic guidance was achieved, an optimal position was achieved under TEE adjustment in 24 patients. In the remaining 41 patients, no optimization, despite TEE guidance, was possible, and the position remained suboptimal. In every patient in whom a poor position (*n* = 12, 8.0%) of the TSP system was achieved under fluoroscopic guidance only, an optimization could be achieved. In three and nine patients, an optimal and suboptimal position, respectively, was achieved under TEE guidance. In the group of patients with a dangerous position (*n* = 24, 16.0%) after fluoroscopic guidance, an optimal position was established after TEE adjustment in 13 patients, and 11 patients were improved to a suboptimal position (Figure 3).

Under TEE guidance, an improvement to an optimal (*n* = 40) or suboptimal (*n* = 20) position could be achieved in a significant proportion of the patients (*p* < 0.001). Overall, an optimal position was achieved in 89 patients (59.3%) after TEE guidance, while a suboptimal position was achieved in 61 patients (40.7%). A poor or dangerous position was not observed after TEE guidance. In comparing the catheter positions attained through fluoroscopic positioning alone with those achieved with TEE-guided positioning, it is noteworthy that the positions guided by TEE are statistically significantly better (*p* < 0.001).

## 4. Discussion

### 4.1. Feasibility and Accuracy

Apart from the TEE intubation, the EP examination can be performed in the same way as without TEE-guided TSP. The intervention typically proceeds during most of the time the TEE is performed. Only during the insertion of the TEE probe can the interventionalist not proceed with the examination. In most procedures, the placement of the TEE probe was successful on the first attempt. Therefore, the TEE-guided TSP does not seem to be time-consuming regarding the procedural duration.

Our study shows that the in-lab time of the echocardiograph is less than 20 min and accounts for less than 20% of the total procedure duration. Nevertheless, to perform TSP-guiding by TEE, an additional person is needed. This can be an impediment to performing TEE-guided TSPs routinely. In the absence of an additional interventionalist, however, it is not necessary to rely on echocardiographic guidance for TSP in general. Intracardiac echocardiography (ICE) could then be used for TSP. With ICE imaging, TSP could be performed by the TSP-performing interventionalist itself without the need for an additional person. However, ICE also presents disadvantages, including the requirement of an additional groin puncture, added costs, and the need for an interventionalist trained in ICE [19,20].

Furthermore, our data suggest that TEE guidance improves the TSP system’s positioning compared to fluoroscopy-guided TSP using LAO (40°) or AP (0°) and RAO (30°) projections without adjusting the fluoroscopic views with the help of EP catheters as additional anatomical landmarks [15]. With the assistance of TEE, the optimal localization of the TSP increased to almost two-thirds of the cohort. This can be the reason for a significant reduction in the procedural duration, as demonstrated in a smaller study [21]. In our study, we exclusively utilized 2D visualization of the interatrial septum. However, it is conceivable that 3D visualization could prove beneficial as it allows for the integration of information from all dimensions into a single view. Further scientific investigations are required to demonstrate the actual advantage of a 3D representation of the septum.

### 4.2. Safety

Regarding the safety of the TEE-guided TSP, one patient (0.7%) experienced a pharyngeal hematoma because of a complicated TEE probe placement. In this case, there were no further sequelae and no need for a surgical intervention. No major complications occurred. In particular, there were no pericardial effusions or pericardial tamponades. These findings are consistent with the published complication rate of TEE-guided TSP reported by Zuercher et al. [22]. From this, it can be deduced that the use of TEE during an EP procedure seems to be safe.

Furthermore, misdirected TSP is a leading cause of cardiac tamponade [23], associated with a mortality rate of 2.3% [24]. Specifically, in 16% of the patients, the position of the TSP system was considered dangerous after fluoroscopic-guided positioning of the TSP system. If the puncture had been performed in this location, it could potentially have had harmful consequences for the patient, possibly resulting in a major complication such as pericardial tamponade. In general, the complication rate of fluoroscopic-guided TSP is reported as 1.3% [25]. There is a relative difference between the number of dangerous positions in the TSP system and the actual reported complication rate. Firstly, it can be partly attributed to the unaffected self-repositioning of the TSP to areas of the least resistance, in our opinion. Secondly, not every misguided punction is necessarily followed by a major complication. This can be due to several safety mechanisms during TSP; for example, left atrial pressure measurements from the transseptal needle tip can prevent the complete advancement of the TSP system in the wrong compartment. If there is no dilatation of the misguided puncture, the puncture channel created by the needle is smaller and typically self-seals without resulting in significant bleeding. TEE-controlled positioning of the TSP system significantly improved the accuracy of the desired TSP localization and effectively prevented poor or dangerous positions of the TSP. In our view, this applies not only to inexperienced but also to experienced interventionalists due to an altered cardiac anatomy. Particularly in older patients with structural heart diseases [20], this can lead to incorrect positioning of the TSP system under purely fluoroscopic control.

### 4.3. Strengths and Limitations

The prospective study underscores the enhanced efficacy of TEE-guided TSP. Assessing the position of the TSP system through both fluoroscopy and TEE in the same patient enables a direct comparison of two identical situations. Nonetheless, the additional safety benefits conferred by TEE-guided TSP are constrained by the absence of a control group. Consequently, there is a pressing need for a larger randomized study to substantiate the safety profile of TEE-guided TSP in EP procedures and ascertain its utility in clinical practice.

## 5. Conclusions

TEE guidance has proven to be a feasible, more accurate, and safe strategy for performing TSP in left atrial EP procedures in comparison to purely fluoroscopy TSP guidance. The localization of the TSP system at the interatrial septum could be optimized from a poor or dangerous position under fluoroscopic guidance in all cases. However, major complications may have been prevented by optimizing the position of the TSP system. Additionally, TEE guidance enabled TSP to be in a desired optimal or at least suboptimal position, providing the electrophysiologist with comfortable handling of the ablation catheters in the left atrium.

## Figures and Tables

**Table 1:** Description of the fluoroscopically achieved TSP system positioning using TEE control during various procedures.

	Optimal Position	Suboptimal Position	Poor Position	Dangerous Position
CB PVI	posteroinferior	midinferior posterosuperior midsuperior midanterior midposterior middle-middle	anterosuperior anteroinferior	extreme posterior extreme anterior extreme inferior extreme superior
RF ablation	middle-middle midinferior	posterosuperior midsuperior midanterior midposterior posteroinferior	anterosuperior anteroinferior	extreme posterior extreme anterior extreme inferior extreme superior
PFA PVI	midanterior	midinferior posterosuperior midsuperior posteroinferior midposterior middle-middle	anterosuperior anteroinferior	extreme posterior extreme anterior extreme inferior extreme superior

CB, cryo-balloon; PFA, pulsed-field ablation; PVI, pulmonary vein isolation; RF, radiofrequency; TEE, transesophageal examination; TSP, transseptal puncture.

**Table 2:** Baseline characteristics.

	Total
Patients, *n*	150
Age, years	71 ± 12
Male, *n* (%)	87 (58.0)
Female, *n* (%)	63 (42.0)
BMI (Body mass index), kg/m^2^	27.4 ± 4.7
Left ventricular ejection fraction, %	53 ± 13
Left atrial diameter, cm	4.6 ± 0.7
Coronary artery disease, *n* (%)	62 (41.3)
Arterial Hypertension, *n* (%)	109 (72.7)
Diabetes mellitus, *n* (%)	25 (16.7)
COPD, *n* (%)	7 (4.7)
OSAS, *n* (%)	12 (8.0)

Values are *n*, *n* (%), mean ± SD; BMI, body mass index; COPD, chronic obstructive pulmonary disease; LVEF, left ventricular ejection fraction; OSAS, obstructive sleep apnea syndrome; SD, standard deviation.

**Table 3:** Procedural characteristics.

	Total	Radiofrequency Ablation	Cryo-Balloon Ablation	Pulsed-Field Ablation
Number, *n* (%)	150	75 (50.0)	38 (25.3)	37 (24.7)
Procedure duration, min	99 ± 45	130 ± 5	74 ± 3	60 ± 2
Fluoroscopy, min	15.0 ± 8.3	12.3 ± 1.0	15.8 ± 1.2	19.5 ± 0.9

Values are *n*, *n* (%), mean ± SD; min, minutes; SD, standard deviation.

**Table 4:** Detailed information on the transesophageal examination.

	Total
TEE intubation attempts, *n*	1.8 ± 1.5
TEE duration, min	17.2 ± 7.6
Minor TEE-associated complication, *n* (%)	1 (0.7)
Major TEE-associated complication, *n* (%)	0
Unsuccessful TEE intubation, *n* (%)	2 (1.3)

Values are *n*, *n* (%), mean ± SD; min, minutes; SD, standard deviation.

## Data Availability

The data presented in this study are available on request from the corresponding author. The data are not publicly available due to data privacy law.
